# Grass species and climatic season impact on *Rhipicephalus microplus* temporal abundance in a tropical region

**DOI:** 10.1002/ps.70941

**Published:** 2026-05-19

**Authors:** Valesca Henrique Lima, Cárita de Souza Ribeiro‐Silva, Salorrane Miranda do Nascimento Pinto, Caio Márcio de Oliveira Monteiro, Pricila Vetrano Rizzo, Fernanda Mara Cunha Freitas, Rafael de Andrade Moral, Gabriel Moura Mascarin, Éverton Kort Kamp Fernandes

**Affiliations:** ^1^ Programa de Pós‐graduação em Ciência Animal Universidade Federal de Goiás Goiânia Brazil; ^2^ Programa de Pós‐graduação em Medicina Tropical e Saúde Pública Universidade Federal de Goiás Goiânia Brazil; ^3^ Instituto de Patologia Tropical e Saúde Pública, Universidade Federal de Goiás Goiânia Brazil; ^4^ Embrapa Arroz e Feijão Santo Antônio de Goiás Brazil; ^5^ Department of Mathematics and Statistics Maynooth University Maynooth Ireland; ^6^ Embrapa Meio Ambiente Jaguariúna Brazil

**Keywords:** cattle tick, climatic data, ecology, forage plants, integrated *Rhipicephalus microplus* management, off‐host ticks

## Abstract

**BACKGROUND:**

The cattle tick *Rhipicephalus microplus* is a major constraint to livestock production in tropical regions, causing substantial economic losses and driving intensive acaricide use. Despite its importance, the role of pasture composition in shaping off‐host tick populations remains poorly understood. This study evaluated whether forage grass species create distinct microenvironments that influence the seasonal abundance and persistence of *R. microplus* larvae in a tropical region.

**RESULTS:**

Larval abundance was higher overall in the dry season than in the rainy season, with a significant interaction between grass and season. Grass cultivars differed markedly during the dry season. Two *Megathyrsus maximus* cultivars supported the highest larval densities and the longest recovery windows, whereas two *Urochloa* sp. cultivars showed the lowest larval densities. Differences in larval abundance were not significant during the rainy season across grasses (*P* = 0.13). Minimum temperature and minimum relative humidity were the most influential microclimatic predictors of larval abundance.

**CONCLUSION:**

The interaction between forage species and seasonal climate influences *R. microplus* population dynamics, particularly under dry conditions. Although pasture composition alone is unlikely to determine infestation levels under grazing conditions, and larval abundance in pasture does not necessarily translate directly into infestation levels on cattle, certain grass cultivars may contribute to microenvironments less favorable for tick survival. These findings highlight the potential of forage species to influence off‐host tick dynamics, acting as a complementary component of the integrated *R. microplus* management. © 2026 The Author(s). *Pest Management Science* published by John Wiley & Sons Ltd on behalf of Society of Chemical Industry.

## Graphical abstract

The interaction between grass species and climatic season shapes the population dynamics of the cattle tick, *Rhipicephalus microplus*. This information highlights the potential of forage species to influence off‐host tick dynamics. Additionally, minimum temperature and minimum relative humidity were the most influential microclimatic predictors of larval abundance in the pasture.

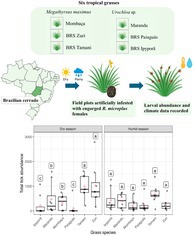

## 1 INTRODUCTION

Ticks and tick‐borne diseases significantly impact livestock health and pose a substantial challenge to cattle production in tropical and subtropical regions worldwide. The cattle tick, *Rhipicephalus microplus* (Acari: Ixodidae), is an economically important ectoparasite and vector of disease agents for bovine babesiosis and anaplasmosis in Brazil, where the national herd comprises ~194 million cattle, accounting for ≈20% of the global cattle population.^1^, ^2^ Estimates indicate that *R. microplus* infestation costs Brazil ≈US$3.2 billion annually.^3^ Additionally, reports of *R. microplus* populations resistant to multiple classes of chemical acaricides in the country are common,^4^, ^5^, ^6^, ^7^ raising concerns about the negative environmental impact of acaricides and their risks for animal and human health.^8^, ^9^ Infestations of *R*. *microplus* impair the sustainability of cattle farming and the competitiveness of global beef production in Brazil and other tropical countries, where most cattle are raised in pastures.^10^ This situation underscores the importance of research in tropical areas focused on how forage species influence the off‐host stages of *R*. *microplus* and should be considered in integrated control strategies.^7^, ^11^, ^12^, ^13^

Managing *R*. *microplus* populations to minimize their impact on cattle health and production is challenging because the nonparasitic life stages comprise ~95% of the entire population of this one‐host tick species in an infested environment.^14^ The off‐host stages include fully engorged and gravid females that dropped off the host, eggs and questing larvae found in pastures. The survival of these free stages is influenced by biotic and abiotic factors.^15^, ^16^ Seasonality is one key factor affecting the nonparasitic stages of *R. microplus* in pastures. Climatic variables such as temperature, humidity, solar radiation and rainfall fluctuate across seasons, and significantly influence tick development, egg incubation, larval survival and activity.^17^ Depending on the time of year, these abiotic factors, which act over several weeks, may create favorable or unfavorable conditions in the field, thereby contributing to the seasonal dynamics of tick populations.^18^, ^19^

Cultivating grasses with morphological and physiological characteristics unfavorable to the nonparasitic stages of *R*. *microplus* may reduce larval populations in pastures, contributing to integrated tick control.^20^, ^21^ Grasses that repel or lack protective effects from environmental factors decrease the odds of survival of nonparasitic stages, thereby minimizing the chances of *R*. *microplus* larvae finding a host.^22^, ^23^ Forage plant morphology may influence the survival of *R. microplus*. Forage plants with erect or clump‐forming growth, such as grasses of the genus *Megathyrsus* (syn. *Panicum*), exposed *R. microplus* larvae to desiccation by allowing light to reach the bases of the grass.^24^ By comparison, stoloniferous grasses that spread laterally through stolons and rhizomes, such as *Cynodon spp*. and some *Urochloa* species (syn. *Brachiaria*), provided advantageous conditions for the development of *R. microplus* larvae.^25^ Furthermore, forage plants such as *U. brizantha* cultivar Marandu, the most widely cultivated forage grass in Brazil, covering an estimated 50 million ha,^26^, ^27^ can exhibit morphological and physiological characteristics of their leaves that are detrimental to tick survival.^28^ Understanding how different grass cultivars interact with seasonal patterns is essential to designing more effective and context‐specific tick control strategies.

Most studies in Brazil assessing the impact of grasses on the survival of the nonparasitic stages of *R. microplus* were conducted by the end of the last century.^29^ Although new grasses were developed for cattle grazing,^26^, ^30^ their effect on the population density of *R. microplus* larvae, particularly cultivars (cv.) of *Urochloa* spp. and *Megathyrsus maximus*, remains unknown. Additionally, grass morphology and architecture can influence microclimatic conditions, making them more or less conducive to tick development, thereby affecting tick population dynamics. Filling this knowledge gap could be crucial in refining pasture management strategies to mitigate the economic impact of *R. microplus* on livestock health and production.

This study tested the hypothesis that *Urochloa* spp. and *Megathyrsus maximum* cultivars provide different microenvironmental conditions and vary in their influence on the abundance and viability of *R. microplus* larvae under tropical conditions. The objectives were to (i) compare the number of active *R. microplus* larvae recovered from different grass cultivars, (ii) identify differences in microenvironmental climate among grasses, and (iii) assess the correlations between microclimatic factors attributed to grasses and the abundance of *R. microplus* larvae. The study was conducted in an experimentally infested pasture during the dry and humid seasons in the Cerrado biome of Brazil (Brazilian savanna).

## 2 MATERIALS AND METHODS

### 2.1 Study site

**Figure 1:**
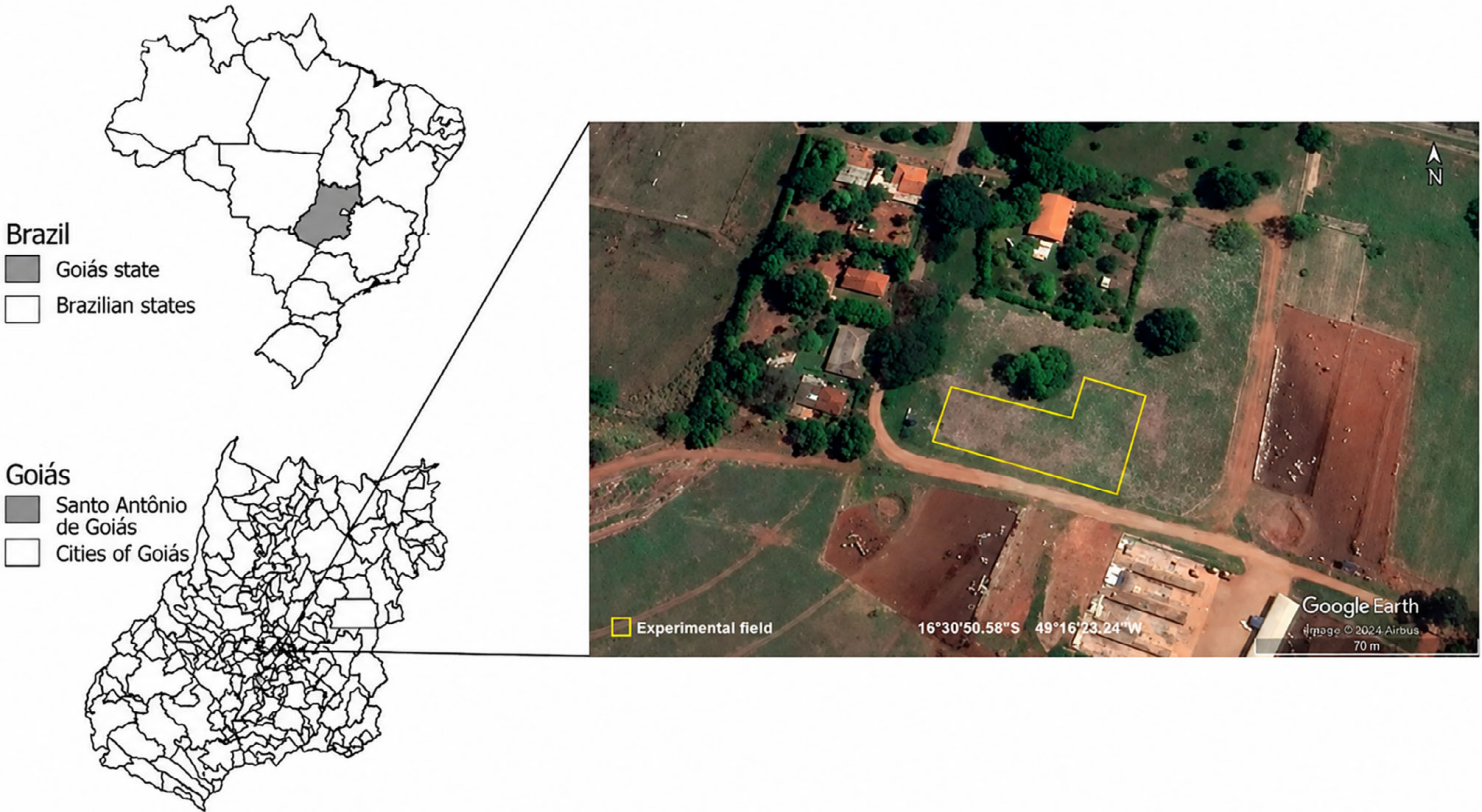
Localization of the study. Brazilian territory, with emphasis on the State of Goiás and the municipality of Santo Antônio de Goiás, and the Experimental area in Embrapa Arroz e Feijão.

The study was conducted between August 2020 and April 2021 at a site in the Agrostological Field of the Regional Nucleus of Embrapa Gado de Leite (16° 30′ 50.1 S; 49° 16′ 33.8 W), located in Embrapa Rice and Beans, municipality of Santo Antônio de Goiás, Goiás state, Brazil (Fig. 1). This location in the Cerrado biome of Brazil, with a Köppen classification for Tropical Savanna (Aw) climate, has a mean annual precipitation of 1.461 mm. The mean annual temperature is 23 ± 0.5 °C,^31^ with well‐defined humid (October to April) and dry (May to September) seasons. The predominant soil type is a Red‐dystrophic Latosol with a clayey texture, characterized by a subperenifolous cerradão phase and flat relief.^32^ The experimental period covered the dry and humid seasons. The dry season trial started on 14 August 2020 and extended until 20 November 2020, whereas the humid season trial was conducted from 19 January 2021 to 23 March 2021.

### 2.2 Field management for grass cultivation

The area was established in 2018 for the cultivation and demonstration of forage plants developed by Embrapa, showcasing technologies related to forage plant management. The field was prepared for grass cultivation by two rounds of plowing and harrowing. The grass seeds were sown in rows with an inter‐row spacing of 45 cm and a density of 5 kg of Pure Live Seeds per hectare (PLS/ha) for *Urochloa* sp. cultivars and 4 kg PLS/ha for *Megathyrsus* sp. cultivars. Basal fertilization was performed with phosphorus pentoxide (P_2_O_5_; 60 kg ha^−1^), potassium oxide (K_2_O; 40 kg ha^−1^), and single top‐dressing fertilization with nitrogen (N; granular urea, 20 kg) and K_2_O (20 kg). The pastures were clipped six times before the study: thrice during the 2018/2019 crop and three times for the 2019/2020 crop. The study site has remained fenced off and inaccessible to bovine grazing since 2018.

The grasses tested in this study were selected based on their productivity and adaptation to Brazilian soils and climate. Selections included three cultivars of *Urochloa* grass—*Urochloa brizantha* cv. Marandu, *U. brizantha* cv. BRS Paiaguás and the hybrid *Urochloa sp*. cv. BRS Ipyporã—and three cultivars of *Megathyrsus maximus* (Jacq.) B.K. Simon & S.W.L. Jacobs (Poales: Poaceae) (syn. *Panicum maximum*): *M. maximus* cv. Mombaça, *M. maximus* cv. BRS Zuri and *M. maximus* cv. BRS Tamani (Table 1). Eight experimental units, each measuring 1 m^2^, hereafter referred to as research plots (48 in total), were established for each of the six selected cultivars. Research plots were delimited by iron stakes and separated from each other by a 1‐m‐wide strip of land devoid of vegetation. An initial inspection of the research plots was conducted to remove weeds and accumulated leaf litter. Grass growing in the research plots was clipped 3 days before the experiment began to create an environment that mimicked natural post‐grazing conditions.^15^, ^33^, ^34^ Following clipping, the height of *Urochloa* cultivars was ~30 cm, and that of *M. maximus* cultivars was ~50 cm.^35^, ^36^, ^37^ Pasture height (cm) was measured with a ruler, and the research plots were clipped every 15 days after the first larval recovery for continued simulation of grazing effects. The same research plots were used during the dry and rainy seasons (Supporting Information, Fig. S1), and all plots experienced the same sunlight and rainfall conditions.

**Table 1:** Grass cultivars evaluated in this study

Species	Cultivar	Origin	Year of release
*Urochloa brizantha*	Marandu	Africa (Zimbabwe)	1984
*Urochloa brizantha*	BRS Paiaguás	Africa (Kenya)	2013
*Urochloa* spp. (hybrid)	BRS Ipyporã	Brazil	2017
*Megathyrsus maximus*	Mombaça	Africa (Tanzania)	1993
*Megathyrsus maximus*	BRS Zuri	Africa (Tanzania)	2014
*Megathyrsus maximus*	BRS Tamani	Brazil	2015

*Note:* The selection included six *Urochloa* spp. and *M. maximus* grasses commonly used for cattle grazing in tropical regions.

### 2.3 Tick exposure

Each research plot was infested once with six *R. microplus* engorged females (Gyn strain),^38^ obtained from artificially infested calves kept at the Escola de Veterinária e Zootecnia of Universidade Federal de Goiás. This population (GYN) is resistant to synthetic pyrethroids, organophosphates, phenylpyrazoles and amitraz.^39^ Engorged female ticks were collected manually from the cattle and transported to the laboratory, washed in distilled water, immersed in 0.1% sodium hypochlorite solution for 3 min, rinsed in distilled water and dried in sterile paper towels. Ticks that weighed ≥150 mg were selected^40^ to compose the 48 groups of six females, each with an equivalent average weight, ensuring that tick groups used to infest the research plots in both seasons averaged similar weights (*P* > 0.05). On the same day (Day 0), six engorged female ticks were released in each research plot close to the grass base. Once released, the females were not disturbed to allow them to complete oviposition. The research plots were inspected weekly after infestation to check for the presence of tick predators, such as birds and ants.

### 2.4 Larval recovery

**Figure 2:**
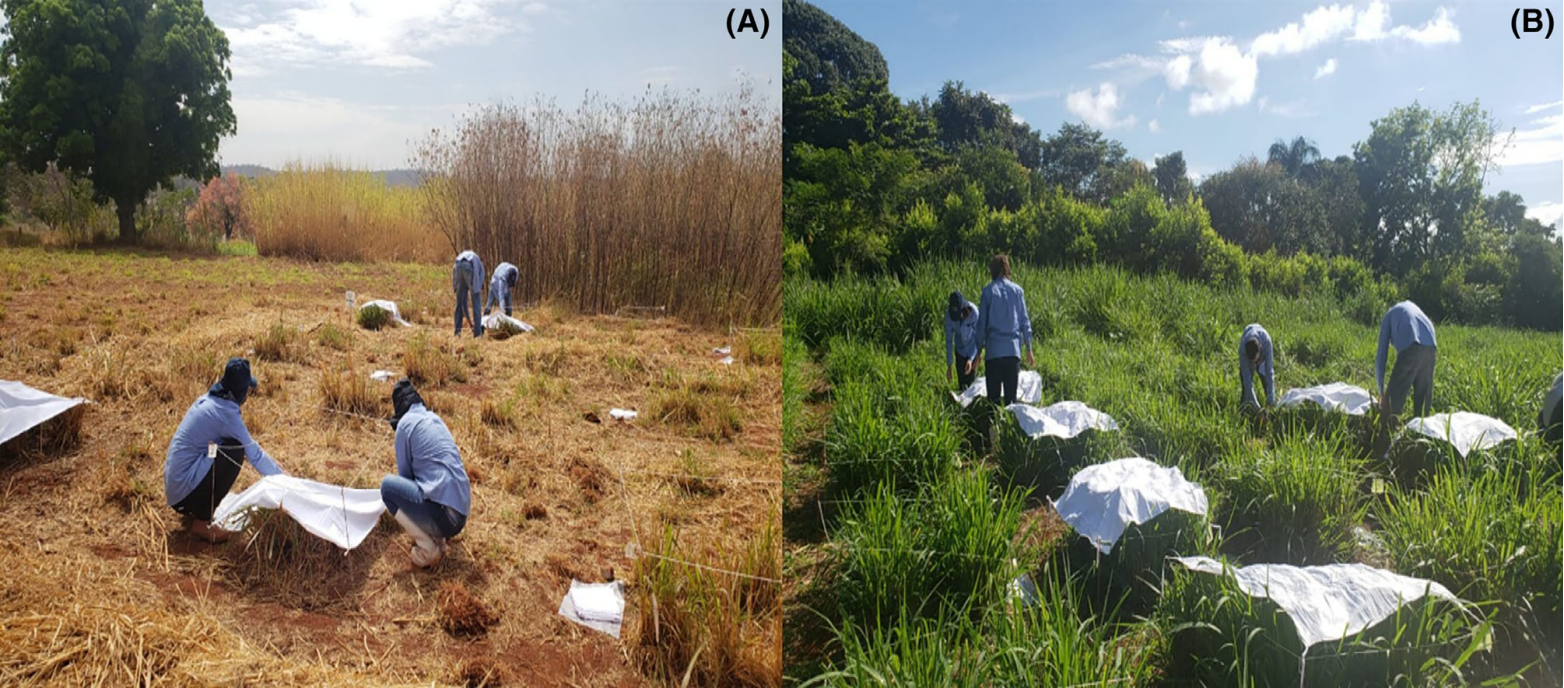
Collection of *Rhipicephalus microplus* larvae from artificially infested research plots using white flannel cloth during the dry (A) and rainy seasons (B).

Thirty days postinfestation (D +30), each research plot was inspected to detect the presence of tick larvae. Upon detection, tick larvae were collected from each research plot at 7‐day intervals, consistently starting at 09:30 h (Brasília‐DF time zone, GMT‐3). Collection continued until no tick larvae were detected in any plot. Tick larvae were collected by placing a white flannel cloth (1.3 m × 1.3 m) on each plot, covering the vegetation, for 15 min^41^ (Fig. 2). Flannel cloths used to collect tick larvae were packed individually in plastic bags labeled with the cultivar, collection date and corresponding research plot number. Plastic bags were frozen at −20 °C, and the tick larvae collected were counted within 3 days. Tick larval counts were conducted under a stereomicroscope using a plastic tip attached to a surgical fluid aspirator (Aspiramax®; Maxima SpA, Kyoto, Japan), with each larva individually counted. The total number of tick larvae recovered from each flannel cloth was recorded.

### 2.5 Assessment of macro‐ and microenvironmental conditions

**Figure 3:**
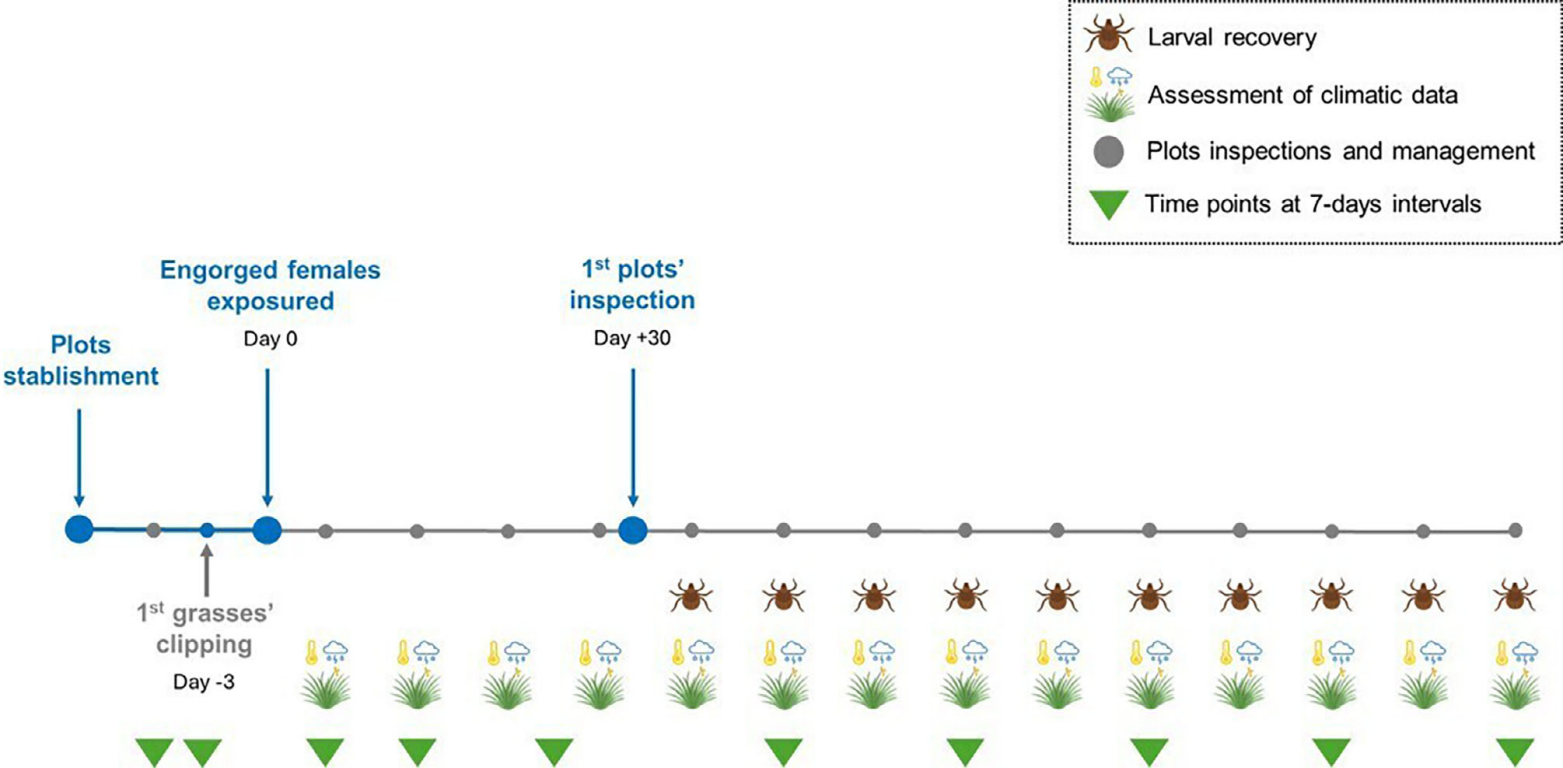
Schematic representation of the experimental design, including a timeline for assessment of environmental conditions. The study period encompassed the dry and rainy seasons, as depicted in Fig. 2.

Daily climate data were obtained from the meteorological station at Embrapa Rice and Beans (located ≈1 km from the study site) throughout the experimental period and used to calculate weekly averages for air temperature, air humidity and air saturation deficit. The averages were analyzed to verify the effects of macroclimatic variables. A schematic representation of the experimental design, including a timeline for plot inspection and management, larval recovery, and assessment of environmental conditions, is presented in Fig. 3.

Because microenvironmental conditions are considered to play a key role in the survival of the nonparasitic life stages of ticks,^42^, ^43^, ^44^ relative humidity (RH) and temperature were measured in research plots using data loggers (UX100‐023A; HOBO® Data Loggers, Bourne, MA, USA). The sensors were placed at the ground level in the center of one randomly selected research plot from the eight replicates for each of the six cultivars. Records obtained at hourly intervals beginning on D0 were used to calculate weekly average minimum and maximum temperature (°C) and RH (%). Moreover, as a measure of the drying power of the atmosphere at a given temperature and RH, the saturation deficit (SD, mmHg) was calculated because it has been shown to modulate the lifespan of ticks in their nonparasitic stages.^42^, ^43^, ^45^ SD was calculated according to the following formula.^42^

$$\mathrm{SD}=\left(1–\mathrm{RH}/100\right)\times 4.9463\times e^{\left(0.0621\;x\;T\right)}$$

where *e* is Euler's number (mathematical constant, ≈2.71828).

### 2.6 Statistical analysis

Initially, to investigate the association between tick numbers and microclimatic variables, a negative binomial generalized additive model (GAM) was fitted. This model included the effects of blocks as fixed, a random plot intercept, the two‐way interaction between season and grass type, thin‐plate regression splines over time per season and per grass type, as well as thin‐plate regression splines over the minimum, mean and maximum values of temperature, RH and SD. The significance of the effects was assessed using chi‐squared (χ^2^) tests, and only the significant climatic variables were retained for further analyses.

Negative binomial GAMs were fitted to the data to assess the effects of the macroclimatic variables, including the impact of grass type, season, and their two‐way interaction, as well as different thin plate regression splines over time per grass type and season, and thin plate regression splines over air temperature and air humidity. We assessed the significance of smooths using χ^2^ tests. To determine whether separate smooths would provide a better fit to the data, we also fitted an alternative model that included unidimensional thin plate regression splines for air temperature and air humidity separately. We calculated the Akaike information criterion (AIC) for both models and selected the model that presented the lowest AIC.

In order to assess the differences in the number of ticks between grass types within each season and the seasons within each grass type, we fitted negative binomial GAMs, including the effects of blocks as fixed, random plot intercepts, grass type and thin‐plate regression splines over time by grass type, minimum temperature and minimum RH. Grass types were then compared through likelihood‐ratio tests between nested models. To analyze differences in the total number of ticks between dry and rainy seasons and among grass types, we fitted negative binomial generalized linear models (GLMs) that included the effects of block and a two‐way interaction between grass type and season. Multiple comparisons were then performed using likelihood ratio tests between nested models.

Goodness‐of‐fit for all fitted models was assessed using normal plots with a simulated envelope.^46^ All GAMs were fitted using the mgcv package^47^ for R software.^48^

### 2.7 Ethics statement

The studies with *R. microplus* were conducted in accordance with the regulations of the Ethics Committee on the Use of Animals of the Universidade Federal de Goiás (UFG, protocol no. 075/18). Access to Brazilian genetic heritage was approved by the Genetic Heritage Management Council (CGen) of Brazil (SisGen protocol no. A420934).

## 3 RESULTS

### 3.1 Grass species and season affecting temporal tick fluctuation

Larval tick numbers were analyzed by grass and by season. Regardless of the grass species, there were 25 169 *R. microplus* larvae collected in total throughout the experimental course during the dry season, whereas 17 697 larvae were collected during the rainy season. The maximum larval recovery period (from the first day of collection to the day all counts reached zero) was 10 weeks in the first trial (starting during the dry period), which lasted longer than initially expected. In the rainy season, recovery lasted 9 weeks. The grass species significantly influenced tick recovery time only during the dry season (χ^2^ = 4100.2, df = 5, *P* < 0.0001), with the most prolonged recovery observed on BRS Tamani (mean 7 weeks) and the shortest recovery on BRS Ipyporã (mean 3.4 weeks) and BRS Paiaguás (mean 3.5 weeks). Peak infestation occurred in the second and third weeks of sampling, with 10 109 larvae during the dry season and 6830 during the rainy season (χ^2^ = 124.41, df = 1, *P* < 0.0001) (Table 2).

**Table 2:** Temporal density of *R. microplus* larvae recovery per plot of different pastures over the weeks in both dry and rainy seasons (from D1 of larvae collection to the day that all counts were zero)

Period	Pasture	Exposition (engorged females)	First larval collection	Last larval collection	Number of larvae per collection/week										Total
					1st	2nd	3rd	4th	5th	6th	7th	8th	9th	10th	of larvae
Dry season	*Urochloa brizantha* cv. Marandu	14 Aug 20	18‐Sep‐20	30 Oct 20	68	2901	408	158	212	50	18	0	0	0	3815
	*Urochloa brizantha* cv. BRS Paiaguás	14 Aug 20	25‐Sep‐20	20 Nov 20	0	603	75	74	70	33	5	1	0	1	862
	*Urochloa* spp. cv. BRS Ipyporã	14 Aug 20	25‐Sep‐20	6 Nov 20	0	841	406	62	338	56	9	2	0	0	1714
	*Megathyrsus maximus* cv. Mombaça	14 Aug 20	18‐Sep‐20	13 Nov 20	6	656	726	567	536	173	37	6	1	0	2708
	*Megathyrsus maximus* cv. BRS Zuri	14 Aug 20	18‐Sep‐20	6 Nov 20	2	4584	1562	990	766	192	93	7	0	0	8196
	*Megathyrsus maximus* cv. BRS Tamani	14 Aug 20	25‐Sep‐20	20 Nov 20	0	524	2775	2039	1500	659	314	47	15	1	7874
Rainy season	*Urochloa brizantha* cv. Marandu	19 Jan 21	26 Feb 21	16 Mar 21	441	566	1437	382	143	200	16	3	0	‐	3188
	*Urochloa brizantha* cv. BRS Paiaguás	19 Jan 21	26 Feb 21	16 Mar 21	72	490	687	192	265	121	8	1	0	‐	1836
	*Urochloa* spp. cv. BRS Ipyporã	19 Jan 21	26 Feb 21	23 Mar 21	581	698	1279	391	240	147	41	46	1	‐	3424
	*Megathyrsus maximus* cv. Mombaça	19 Jan 21	26 Feb 21	16 Mar 21	128	498	498	305	87	222	5	8	0	‐	1751
	*Megathyrsus maximus* cv. BRS Zuri	19 Jan 21	26 Feb 21	23 Mar 21	263	543	544	378	198	101	9	9	1	‐	2046
	*Megathyrsus maximus* cv. BRS Tamani	19 Jan 21	26 Feb 21	23 Mar 21	279	1283	2385	537	548	360	38	19	3	‐	5452

**Figure 4:**
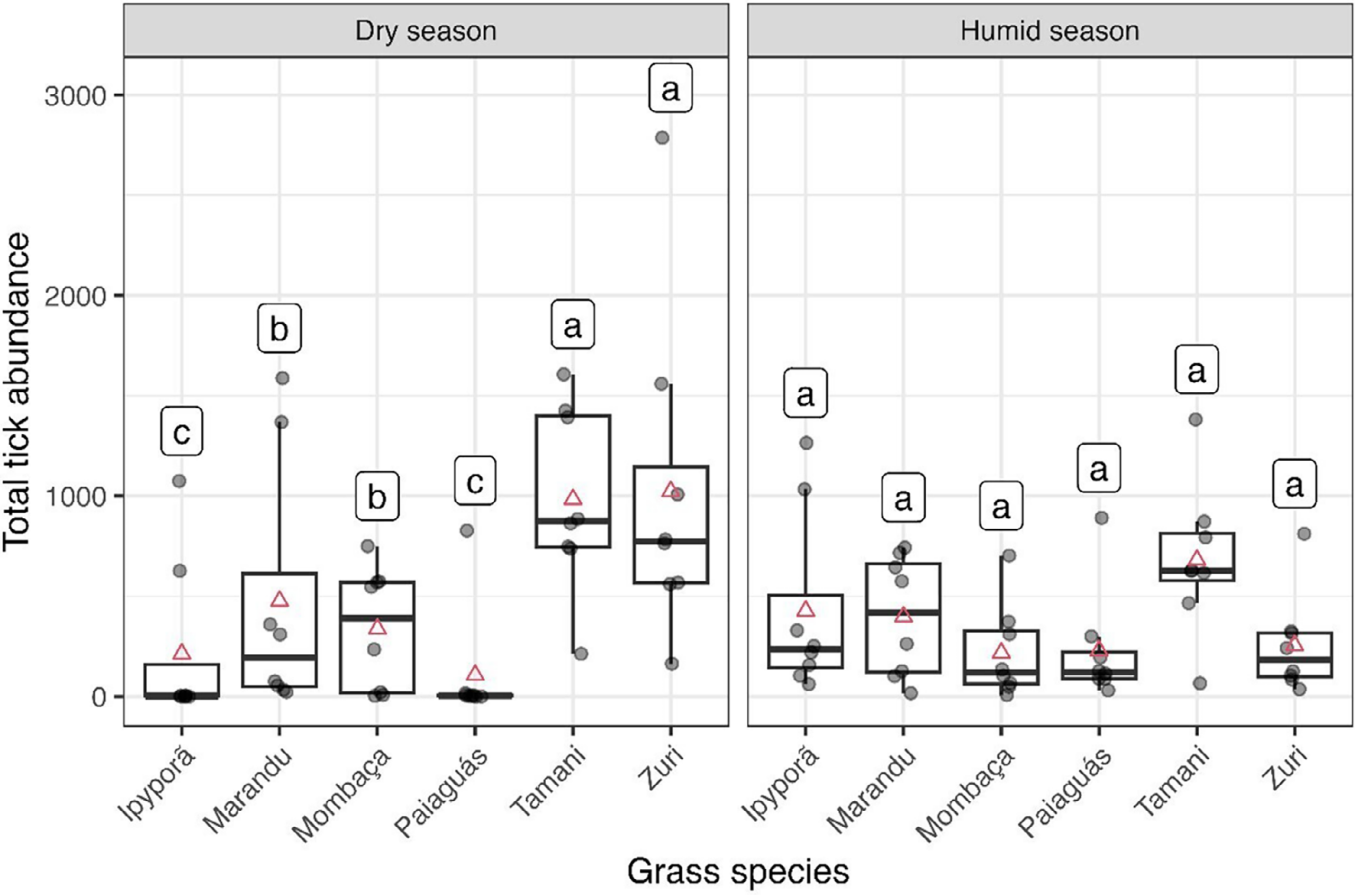
Total abundance of *R. microplus* larvae accumulated across 10 weeks on six different grass species recorded in two seasons (dry and rainy) in the Central West of Brazil. Comparisons were performed among the grasses within each season. The length of the box represents the IQR. The horizontal line within the box represents the median. The whiskers represent the 1.5 IQR of the 25th quartile or the 1.5 IQR of the 75th quartile. The shaded dots represent the raw data. Means (red triangle) followed by distinct letters indicate significant differences in the number of larvae collected in each pasture type (*P* < 0.05) in each season.

The interaction between grass species and climatic season played a significant role in fluctuations in larval density, resulting in distinct patterns observed during the dry and rainy seasons. Our model indicated that larval density was significantly affected by the interaction between grass species and season (χ^2^ = 20.98, df = 5, *P* = 0.0008). Across all observations (8 plots × 10 weeks = 80 per pasture), the larval density was remarkably higher in the dry season than in the rainy season (χ^2^ = 5.63, df = 1, *P* = 0.017). The highest tick density during the dry season was found on BRS Tamani (mean ± SE, 984 ± 163 larvae/m^2^) and BRS Zuri (mean ± SE, 1024 ± 289 larvae/m^2^), whereas the lowest densities occurred on BRS Paiaguás (mean ± SE, 108 ± 103 larvae/m^2^) and BRS Ipyporã (mean ± SE, 214 ± 145 larvae/m^2^) (χ^2^ = 63.76, df = 5, *P* < 0.0001). By contrast, during the rainy season, there were no significant differences for tick larval density between grass species (χ^2^ = 8.51, df = 5, *P* = 0.13), although numerically the BRS Tamani maintained the highest mean tick density among *M. maximus* grasses (mean ± SE, 682 ± 132 larvae/m^2^), followed by BRS Ipyporã (mean ± SE, 428 ± 162 larvae/m^2^), whereas Mombaça (219 ± 82.8 larvae/m^2^) and BRS Paiaguás (230 ± 98.8 larvae/m^2^) exhibited the lowest densities (Fig. 4).

**Figure 5:**
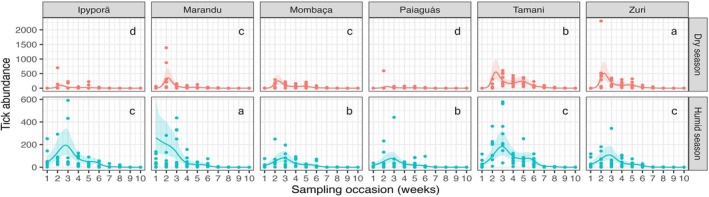
Temporal fluctuation of *R. microplus* larvae recorded from eight plots of each grass in different seasons during 10 weeks. Same letters indicate no significant differences in the number of larvae collected among grasses during the dry or rainy season (*P* > 0.05). Solid lines represent the fitted curves along with raw data as dots.

A closer look at tick abundance accumulated across 10 sampling weeks revealed significant variations among grass species, but these differences diminished during the rainy season. Grass species significantly influenced tick abundance during the dry season (χ^2^ = 23.397, df = 5, *P* = 0.00028), but no differences were detected during the rainy season (χ^2^ = 10.699, df = 5, *P* = 0.0577). BRS Zuri supported higher tick numbers in the dry season compared to the rainy season, while BRS Paiaguás and BRS Ipyporã showed increased tick loads in the rainy season. Statistical analysis revealed no significant differences in tick density for grasses Marandu (χ^2^ = 0.56, df = 1, *P* = 0.47), Mombaça (χ^2^ = 0.001, df = 1, *P* = 0.973) and BRS Tamani (χ^2^ = 1.52, df = 1, *P* = 0.218) (Fig. 5).

Overall, BRS Tamani was the most suitable grass species for tick larvae infestation throughout the year under the given field experimental conditions. Its high tick recovery and density suggest that it is particularly favorable for *R. microplus* larvae, irrespective of the season. Conversely, grasses such as Mombaça and BRS Paiaguás consistently exhibited lower tick loads, making them less suitable for larvae.

### 3.2 Macro‐ and microclimatic factors affecting temporal tick fluctuation

**Figure 6:**
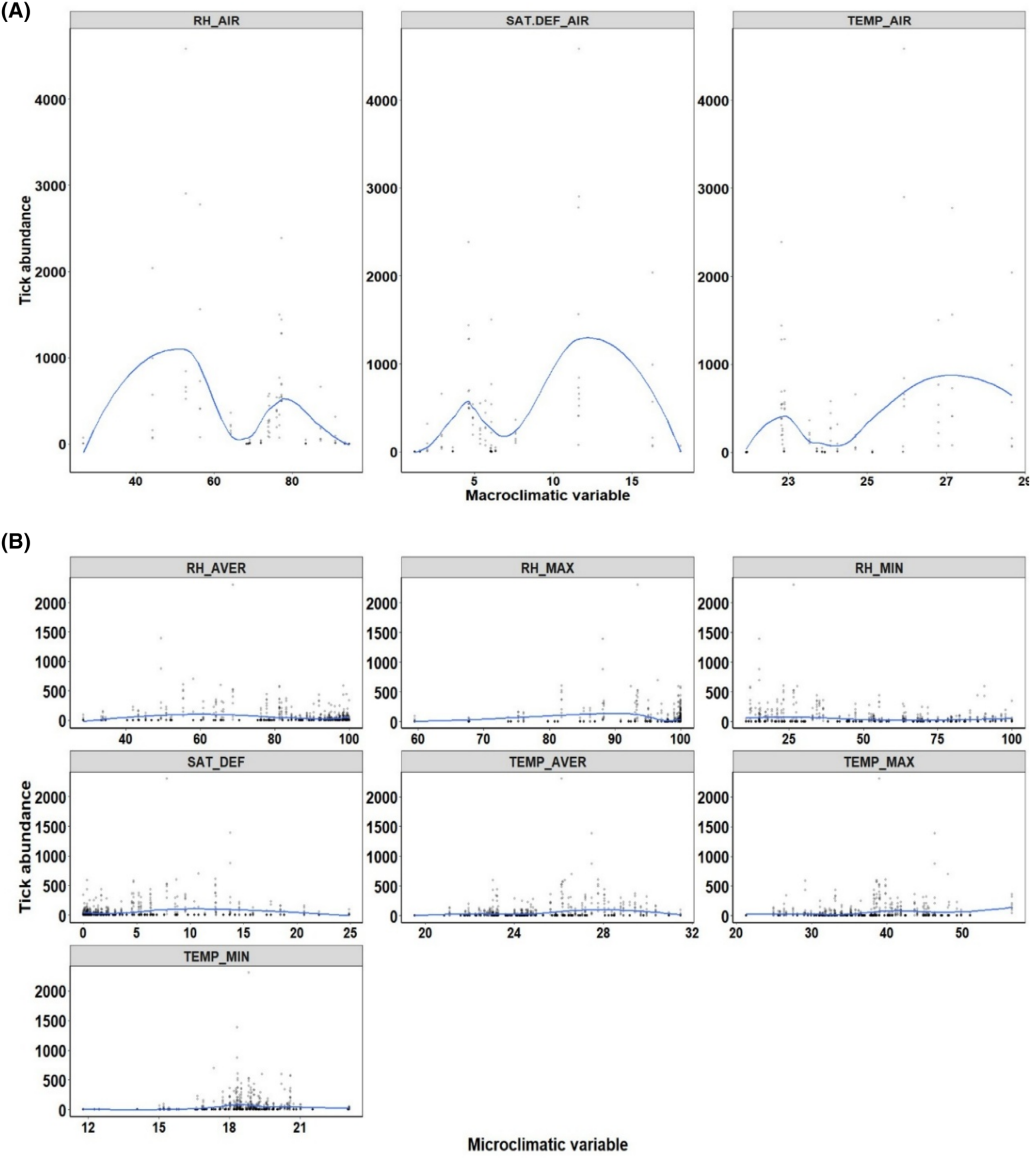
Nonlinear relationship of tick abundance (larvae recovered in field plots) retrieved from all grass species during two seasons (dry and rainy) with macroclimatic (A) and microclimatic variables (B). RH_AVER, average RH; RH_MAX, maximum RH; RH_MIN, minimum RH; SAT_DEF, SD; TEMP_AVER, average temperature; TEMP_MAX, maximum temperature; TEMP_MIN, minimum temperature; RH_AIR, RH of the air: SAT_DEF.AIR, SD of the air; TEMP_AIR, air temperature.

Analyzing the macroclimatic factors (Fig. S2) impacting the tick population dynamics, our model highlighted that only air RH (χ^2^ = 34.27, df = 4.13, 4.40, *P* < 0.0001) significantly influenced tick abundance (Fig. S3). Densities of *R. microplus* larvae according to the macroenvironmental conditions during the experimental period are described in Fig. 6(A). Weekly ground‐level temperature, RH and SD inside the plots during all experimental time (from the period when engorged females were exposed to each grass plot to the end of larvae collections) are depicted in Table S1.

Regarding microclimatic factors, *R. microplus* ticks exposed to grasses during the dry season experienced ground‐level temperatures between 16.5 °C and 31.5 °C, and RH values ranged greatly from 28.7% to 98.2% (Table 3). The association of both parameters (temperature and RH) revealed mean SDs of 6 and 13 mmHg during the season. The observed variability in microenvironment data may be attributed to rainfall events that occurred during the first trial. However, ticks exposed to different grasses during the rainy season lived at temperatures between 15.7 °C and 26.7 °C and RH between 75% and 100%, resulting in SD from 0.8 to 3.1 mmHg. Among microclimatic factors, differences in mean temperature were significant only between *M. maximus* Mombaça and BRS Tamani, during the rainy season (*P* < 0.0279), with RH values also exhibiting significant differences during the rainy season (*P* < 0.0002). Interestingly, RH in *Urochloa brizantha* cv. Marandu differed from BRS Tamani in both seasons (*P* < 0.0099 for the dry season and *P* < 0.0095 for the rainy season); however, SD values were similar (*P* < 0.0562 and *P* < 0.2413).

**Table 3:** Mean values of ground‐level temperature (°C), RH (%) and SD deficit (mmHg) in *Megathyrsus maximus* and *Urochloa* plots

Period	Pasture		Microclimatic conditions	
		Temperature (°C)	RH (%)	SD (mmHg)
Dry season	*Urochloa brizantha* cv. Marandu	26.5^a^	52.4^b^	12.9^ab^
	*Urochloa brizantha* cv. BRS Paiaguás	25.7^a^	67.4^ab^	8.20^b^
	*Urochloa* spp. cv. BRS Ipyporã	26.1^a^	62.2^ab^	9.92^ab^
	*Megathyrsus maximus* cv. Mombaça	26.2^a^	66.0^ab^	8.89^ab^
	*Megathyrsus maximus* cv. BRS Zuri	25.9^a^	70.2^ab^	7.51^ab^
	*Megathyrsus maximus* cv. BRS Tamani	25.1^a^	75.5^a^	6.04^b^
Rainy season	*Urochloa brizantha* cv. Marandu	23.5^ab^	95.1^ab^	1.070^c^
	*Urochloa brizantha* cv. BRS Paiaguás	24.5^ab^	95.0^a^	1.133^a^
	*Urochloa* spp. cv. BRS Ipyporã	23.6^ab^	90.2^bc^	2.047^bc^
	*Megathyrsus maximus* cv. Mombaça	23.2^b^	96.3^a^	0.823^ab^
	*Megathyrsus maximus* cv. BRS Zuri	23.6^ab^	96.2^a^	0.827^c^
	*Megathyrsus maximus* cv. BRS Tamani	24.8^a^	86.6^c^	3.250^bc^

*Note:* The values correspond to the entire experimental period, from the exposure of *Rhipicephalus microplus* engorged females to the last collection of larvae. Means followed by the same letters in the same column do not differ significantly (*P* < 0.05). Comparisons were made among the grasses in each season.

Statistically, amongst the microclimatic factors attributed to the morphological characteristics of each grass species, our model indicated that only minimum temperature (χ^2^ = 24.51, df = 4.39, 5.38, *P* = 0.00024) and minimum RH (χ^2^ = 9.092, df = 1, 1, *P* = 0.00257) significantly influenced tick abundance (Fig. S4). Weekly variation in minimum temperature and minimum RH in plots of the forage grass cultivars is shown in Fig. S5. Densities of *R. microplus* larvae according to the microenvironmental conditions during the experimental period are described in Fig. 6(B).

## 4 DISCUSSION

Our results highlight that forage grasses can influence the microenvironment experienced by off‐host stages of *R. microplus*, affecting larval abundance and persistence under field conditions. The results reported here showed that temperature and humidity in the microenvironments of pasture plots artificially infested with *R*. *microplus* varied among and between cultivars of clump‐forming and stoloniferous grasses. These differences were associated with variation in larval abundance, which should be interpreted as larval recovery or availability under the evaluated conditions rather than a direct measure of absolute survival. Seasonal conditions in the Brazilian Cerrado biome also modulated microclimatic characteristics, with higher numbers of *R*. *microplus* larvae surviving during the dry season compared to the rainy season, as observed during the evaluated months.

The elevated number of *R. microplus* larvae recovered during the dry season in this study confirms previous observations in the same region, indicating that local climatic conditions sustain significant tick activity even in periods of scarce rainfall. Nicaretta *et al*.^18^ reported that the temperature regime in Goiânia, Goiás state, favors the development of nonparasitic stages during the dry season. Although a cumulative effect of residual larvae from earlier generations may have contributed to larval peaks in those studies, the sustained larval presence and delayed decline observed in our trial may be partly attributable to rainfall events during the experimental period. Notably, the first recorded precipitation (0.8 mm) occurred in the Week 6 of the experiment, which corresponded with the second week of sampling larvae. This coincided with a slight decrease in temperature, an increase in RH, and a reduction in the SD at the soil level (Table S1, see weeks 5 and 6). These conditions are known to favor larval survival and activity.^17^ Rainfall, however, plays a dual role: its impact on tick populations depends heavily on the amount, frequency, and distribution of precipitation. Although moderate rainfall can indirectly support larval viability by enhancing microclimatic conditions,^49^, ^50^, ^51^ excessive or erratic precipitation may be detrimental, disrupting egg development, displacing larvae or impairing host‐seeking behavior.^17^ These findings underscore the importance of considering microenvironmental variability within seasonal contexts when assessing tick population dynamics in tropical areas like the Cerrado biome.

The higher densities reported herein for *R. microplus* larvae found on *Megathyrsus maximus* cv. BRS Tamani in both seasons and on *M. maximus* cv. BRS Zuri during the rainy season differ from previous reports, which suggest that clump‐forming grasses generally offer less favorable conditions for tick survival. *Urochloa brizantha* cv. Marandu also harbored high larval densities in our study, although it was reported to be lethal to *R. microplus* larvae.^28^ Barros and Evans^28^ reported that Marandu could retain *R. microplus* on its leaf surfaces, resulting in mortality rates >70%. However, it is crucial to note that the white flannel dragging method^41^ employed here captures only active larvae and thus does not reflect mortality that may have occurred earlier. Therefore, differences among grasses are more appropriately interpreted as differences in larval availability in the vegetation at the time of sampling, rather than a direct measure of absolute survival. This distinction is important for the biological interpretation of larval abundance under the evaluated field conditions.

Larval abundance in all the grasses tested in this study is likely to reflect not only larval densities, but also the cumulative effects of environmental conditions and grass characteristics on earlier tick developmental stages. Although larvae were sampled weekly with limited environmental exposure, engorged females remained on the pasture for ≥10 days for oviposition, and the eggs required ≈27 days to hatch.^19^, ^52^, ^53^, ^54^ These tick stages living off the host, therefore, experienced prolonged exposure to the microclimatic and physical features of each grass species. Thus, the influence of grasses on larval abundance may be indirect, mediated by effects on female survival, oviposition success and egg viability. Further research is needed to disentangle the relative contributions of each life stage to understand better how pasture characteristics shape *R. microplus* population dynamics.

Temperature and humidity impact the survival of ticks when they are off the host.^17^ Ground‐level microclimatic measurements are considered more representative of the actual conditions experienced by ticks, as they accurately reflect the influence of vegetation cover.^55^ By combining field‐level microclimatic data with meteorological records from a nearby weather station, we evaluated how these variables influence larval abundance. This approach revealed significant differences in temperature, RH and SD across grass cultivars (see Table S1). Our model indicated that only minimum temperature and minimum RH from ground‐level microclimate data significantly influenced the number of larvae recovered. By contrast, only the air RH showed a significant effect among the macroclimatic variables tested. Although RH > 70% is considered necessary for the survival of engorged females and hatching of *R. microplus* larvae,^56^, ^57^, ^58^ we observed high numbers of viable larvae even in pastures such as *U. brizantha* cv. Marandu, where the weekly average RH was ≈40% during the initial weeks of the experiment (see Table S1). In this context, it is important to underscore that ground‐level microclimatic measurements were obtained from a single plot per cultivar, which may not fully capture within‐cultivar spatial variability in microenvironmental conditions. This limitation should be considered when interpreting the strength of associations between microclimatic variables and larval abundance.

Saturation deficit was not a good predictor of *R. microplus* larval abundance in our multivariate model. Although SD – defined as a function of temperature and RH – is widely recognized as an indicator of air dryness and a key factor influencing tick survival and host‐seeking behavior,^45^, ^59^ our data did not support a direct association with larval density in the field. Teel^45^ reported that high SD (>7 mmHg) negatively affected egg hatchability in *R. microplus* and *R. annulatus*, which correlated with increased egg mass weight loss and reduced hatch percentages. By contrast, our study recorded SD > 12 mmHg in some plots, particularly on *U. brizantha* cv. Marandu during the dry season, without a corresponding decline in larval abundance. However, SD in our multivariate model was inversely correlated with RH, which showed a significant effect on tick abundance. Hence, minimum humidity apparently is a key factor in determining the abundance of *R. microplus* larvae in pastures. These observations indicate that differences in tick abundance and survival influencing tick population density among grasses involve factors beyond microclimatic variables.

Pasture plants with known ‘anti‐tick’ effect attributed to trichomes that physically trap ticks or secrete secondary metabolites with repellent or toxic effects^60^, ^61^ include *Melinis minutiflora* ^28^, ^62^, ^63^ and legumes of the genus *Stylosanthes*.^33^, ^60^ The cultivars assessed in this study exhibit varied trichome densities and distributions (see Embrapa, Pasto Certo App https://www.pastocerto.com/ for details), which may have influenced larval recovery. However, the presence of trichomes alone does not fully explain the differences in larval density. For instance, BRS Ipyporã, despite having dense trichomes, supported larval densities similar to BRS Tamani, which lacks trichomes. Likewise, Marandu (medium to high trichome density) exhibited larval densities comparable to other low‐trichome grasses such as Mombaça.

Larval densities in the field do not directly equate to infestation levels on cattle; thus, caution is required when extrapolating larval abundance in pasture to actual infestation levels in grazing cattle. Factors related to host susceptibility such as breed, age and immunity,^21^, ^64^, ^65^ and stocking rate (the number of animals per unit area) also influence tick burden.^52^ High‐density grazing systems increase the probability of larva–host contact, which results in heavier tick infestations.^11^, ^52^, ^65^, ^66^ According to the Brazilian Agricultural Research Corporation (Embrapa, Pasto Certo App), stocking rates vary among grass species and seasons. BRS Tamani, for example, supports lower stocking rates (1.6 AU ha^−1^ in the dry season and 3.2 AU ha^−1^ in the rainy season), which could reduce opportunities for larvae to find hosts, but the pasture appears advantageous for higher larval survival or development. Conversely, BRS Paiaguás exhibited lower larval abundance, but this may lead to increased tick loads owing to higher animal densities. However, these findings should be interpreted with caution when extrapolating to grazing systems, as the experiment was conducted in artificially infested plots and larval abundance in pasture does not necessarily translate directly into infestation levels on cattle under free‐grazing conditions.

Combining lower‐risk grass with appropriate stocking rates may reduce tick loads while maintaining cattle productivity. However, the selection of forage species and rotational grazing schemes is driven primarily by the herd's nutritional requirements and the system's economic viability, and tick control strategies should not compromise these foundational aspects. Effective pasture‐based tick control requires an integrated approach balancing forage characteristics, environmental conditions, and herd management. Our findings expand understanding of how pasture characteristics influence the ecology of *R. microplus* under experimental conditions. However, further studies in naturally infested grazing systems are required to determine how these microenvironmental effects translate into tick burdens on cattle. In this context, forage selection should be viewed as a complementary, rather than a standalone component of integrated tick management. Effective pasture‐based tick control will likely depend on integrating forage characteristics with host management, environmental conditions, and other control strategies.

Overall, our research highlights opportunities for forage breeding programs to develop cultivars specifically engineered for tick management, thereby reducing reliance on chemical acaricides and promoting environmentally sustainable practices. Some of these practices, such as the use of microorganisms for biological control, including entomopathogenic fungi and nematodes applied to soil,^32^, ^67^, ^68^ can be strengthened by a clear understanding of how pastures influence the nonparasitic phases of *R. microplus*. Given Brazil's prominent role as the world's leading exporter of tropical forage seeds, primarily based on the genera *Urochloa* and *Megathyrsus*, these findings also may have broader implications for cattle production systems in other tropical regions.

## 5 CONCLUSIONS

This study demonstrates that stoloniferous and clump‐forming grasses, specifically *Urochloa* spp. and *M. maximus*, can create contrasting microenvironments that influence the abundance and survival of *R. microplus* larvae under experimental field conditions. Results revealed cultivar‐dependent variations in larval recovery, which were partially explained by microclimatic parameters, including temperature and RH, but not SD, as well as seasonal patterns in the Cerrado biome. These findings highlight the role of pasture structure and microenvironmental variability in tick ecology, suggesting that grass selection may contribute to integrated tick management when combined with other control strategies. However, as this study was conducted in artificially infested plots, further research under natural grazing conditions is needed to determine how these effects translate into infestation levels on cattle. The knowledge generated by correlating grass traits and climatic factors with tick dynamics may guide future breeding programs to enhance desirable characteristics in forage plants. Future studies also should explore how pasture type interacts with other control tools, such as biological agents, to develop sustainable strategies that reduce reliance on chemical acaricides in tropical and subtropical cattle production systems.

## FUNDING INFORMATION

This research was supported by grants from the Conselho Nacional de Desenvolvimento Científico e Tecnológico (CNPq) of Brazil (420 413/2023–5), and the Instituto Nacional de Ciência e Tecnologia em Bioinsumos Inovadores (INCT, CNPq 406 803/2022–6). CNPq provided the grant PQ 308375/2022–0 for É.K.K.F. and a PhD scholarship for V.H.L. The Coordenação de Aperfeiçoamento de Pessoal de Nível Superior (CAPES) of Brazil provided PhD scholarships for C.S.R‐S. and S.M.N.P. (finance code 001).

## CONFLICT OF INTEREST

The authors declare that they have no conflicts of interest.

## AUTHOR CONTRIBUTIONS

**Valesca Henrique Lima:** Conceptualization, investigation, methodology, formal analysis, visualization, writing – original draft, writing – review and editing, project administration, visualization; **Cárita de Souza Ribeiro‐Silva:** Conceptualization, investigation, writing – original draft, writing – review and editing; **Salorrane Miranda do Nascimento Pinto:** Investigation, writing – review and editing; **Caio Márcio de Oliveira Monteiro:** Writing – review and editing; **Pricila Vetrano Rizzo:** Investigation, resources, writing – review and editing; **Fernanda Mara Cunha Freitas:** Resources, writing – review and editing; **Rafael de Andrade Moral:** Formal analysis, validation, writing – review and editing; **Gabriel Moura Mascarin:** Formal analysis, validation, visualization, writing – original draft, writing – review and editing; **Éverton Kort Kamp Fernandes:** Project administration, conceptualization, methodology, validation, visualization, funding acquisition, writing – review and editing.

## Supporting information


**Fig. S1.** Visual comparison of forage grass cultivars during the dry and rainy seasons. Photographs display six forage cultivars under field conditions, highlighting seasonal differences in vegetative vigor, canopy structure and ground coverage. The panel includes cultivars of *Megathyrsus maximus*: cv. Mombaça, BRS Zuri and BRS Tamani, also *Urochloa brizantha*: cv. Marandu, BRS Paiaguás and *Urochloa* spp. cv. BRS Ipyporã. All plots were delimited using 1 m^2^ quadrats to standardize visual comparisons. During the dry season, reduced green biomass and partial senescence were evident across all cultivars. By contrast, during the rainy season, increased biomass and greener foliage were observed.
**Fig. S2.** Macroclimatic data recorded during the study period. Maximum and minimum daily temperature (°C), global solar radiation (MJ m^−2^) and precipitation (mm day^−1^) during the dry and wet season. Data were collected daily at a meteorological station located ≈1 km from the experimental area and include information since the insertion of engorged females (D0) until the last day of collecting *R. microplus* larvae (see Table 2 for information on specific dates).
**Fig. S3.** Macroclimatic variables influencing tick abundance. Partial effects of macroclimatic variables on tick abundance, estimated using GAMs. The left panel shows the effect of air temperature (TEMP_AIR) and the right panel air RH (RH_AIR). Solid lines represent the estimated smooth functions, and shaded areas denote 95% CIs.
**Fig. S4.** Microclimatic variables influencing tick abundance. Partial effects of microclimatic variables on tick abundance over time, as estimated by GAMs. Each panel shows the smoothed effect of a specific predictor variable on tick abundance while holding other variables constant. Curves represent estimated effects with 95% CIs (gray shading), considering interactions with different forage grass cultivars (Ipyporã, Marandu, Mombaça, Paiaguás, Tamani and Zuri), seasons (dry and rainy) and climatic variables (minimum, average and maximum temperature; minimum, average and maximum RH; and SD).
**Fig. S5.** Weekly variation in microclimatic conditions (minimum temperature and RH) recorded at ground level in plots of six forage grass cultivars during the dry and rainy seasons. Orange lines represent minimum temperature (TEMP, °C) and blue lines minimum RH (%). Data were collected near the soil surface for each cultivar: *Urochloa brizantha* (cv. Marandu and BRS Paiaguás), *Urochloa* spp. (cv. BRS Ipyporã) and *Megathyrsus maximus* (cv. Mombaça, BRS Zuri and BRS Tamani).

**Table S1.** Weekly mean ground level temperature (°C), RH (%) and SD (mmHg) inside the plots during the complete experimental period (from the period engorged females were exposed to each grass to the end of larvae collections).

## Data Availability

The data that support the findings of this study are available from the corresponding author upon reasonable request.
