# Supplementary material for: Grass species and climatic season impact on Rhipicephalus microplus temporal abundance in a tropical region
Source: Pest Manag Sci. 2026 May 19;82(9):8950–62. doi: 10.1002/ps.70941 (PMC13453270; doi:10.1002/ps.70941)
Supplement: Supplementary file 1 [file PS-82-8950-s001.docx]

**Supplementary figures**

**Supplementary Fig. S1.** Visual comparison of forage grass cultivars during the dry and rainy seasons. Photographs display six forage cultivars under field conditions, highlighting seasonal differences in vegetative vigor, canopy structure, and ground coverage. The panel includes cultivars of *Megathyrsus maximus*: cv. Mombaça, BRS Zuri, and BRS Tamani, also *Urochloa brizantha*: cv. Marandu, BRS Paiaguás, and *Urochloa* spp. cv. BRS Ipyporã. All plots were delimited using 1 m² quadrats to standardize visual comparisons. During the dry season, reduced green biomass and partial senescence were evident across all cultivars. In contrast, during the rainy season, increased biomass and greener foliage were observed.

**Supplementary Fig. S2** Macroclimatic data recorded during the study period. Maximum and minimum daily temperature (°C), global solar radiation (MJ m^-2^), and precipitation (mm day^-1^) during the dry and wet season. Data were collected daily at a meteorological station located approximately 1 km from the experimental area and include information since the insertion of engorged females (D0) until the last day of collecting *Rhipicephalus microplus* larvae (see Table 2 for information on specific dates)

**Supplementary Fig. S3** Macroclimatic variables influencing tick abundance. Partial effects of macroclimatic variables on tick abundance, estimated using generalized additive models (GAMs). The left panel shows the effect of air temperature (TEMP_AIR), the right panel shows the effect of air relative humidity (RH_AIR). Solid lines represent the estimated smooth functions, and shaded areas denote 95% confidence intervales.

**Supplementary Fig. S4** Microclimatic variables influencing tick abundance. Partial effects of microclimatic variables on tick abundance over time, as estimated by generalized additive models (GAMs). Each panel shows the smoothed effect of a specific predictor variable on tick abundance while holding other variables constant. Curves represent estimated effects with 95% confidence intervals (gray shading), considering interactions with different forage grass cultivars (Ipyporã, Marandu, Mombaça, Paiaguás, Tamani, and Zuri), seasons (dry and rainy), and climatic variables (minimum, average, and maximum temperature; minimum, average, and maximum relative humidity; and saturation deficit).

**Supplementary Fig. S5.** Weekly variation in microclimatic conditions (minimum temperature and relative humidity) recorded at ground level in plots of six forage grass cultivars during the dry and rainy seasons. Orange lines represent minimum temperature (TEMP, °C) and blue lines represent minimum relative humidity (RH, %). Data were collected near the soil surface for each cultivar: *Urochloa brizantha* (cv. Marandu and BRS Paiaguás), *Urochloa* spp. (cv. BRS Ipyporã), and *Megathyrsus maximus* (cv. Mombaça, BRS Zuri, and BRS Tamani).
